# Performance of the EQ-5D-Y Interviewer Administered Version in Young Children

**DOI:** 10.3390/children9010093

**Published:** 2022-01-10

**Authors:** Razia Amien, Desiree Scott, Janine Verstraete

**Affiliations:** 1 Division of Physiotherapy, Faculty of Health and Rehabilitation Sciences, University of Cape Town, Cape Town 7701, South Africa; des.scott@uct.ac.za; 2 Division of Pulmonology, Department of Paediatric and Child Health, University of Cape Town, Cape Town 7701, South Africa; Janine.verstraete@uct.ac.za

**Keywords:** children, youth, interviewer-administered, self-complete, health-related quality of life, EQ-5D-Y

## Abstract

(1) Background: An estimated 78% of South African children aged 9–10 years have not mastered basic reading, therefore potentially excluding them from self-reporting on health-related outcome measures. Thus, the aim of this study was to compare the performance of the EQ-5D-Y-3L self-complete to the newly developed interviewer-administered version in children 8–10 years. (2) Methods: Children (*n* = 207) with chronic respiratory illnesses, functional disabilities, orthopaedic conditions and from the general population completed the EQ-5D-Y-3L self-complete and interviewer-administered versions, Moods and Feelings Questionnaire (MFQ) and Faces Pain Scale-Revised (FPS-R). A functional independence measure (WeeFIM) was completed by the researcher. (3) Results: The 8-year-olds had significantly higher missing responses (x^2^ = 14.23, *p* < 0.001) on the self-complete version. Known-group and concurrent validity were comparable across dimensions, utility and VAS scores for the two versions. The dimensions showed low to moderate convergent validity with similar items on the MFQ, FPS-R and WeeFIM with significantly higher correlations between the interviewer-administered dimensions of Mobility and WeeFIM mobility total (z = 1.91, *p* = 0.028) and Looking After Myself and WeeFIM self-care total (z = 3.24, *p* = 0.001). Children preferred the interviewer-administered version (60%) (x^2^ = 21.87, *p* < 0.001) with 22% of the reasons attributed to literacy level. (4) Conclusions: The EQ-5D-Y-3L interviewer-administered version is valid and reliable in children aged 8–10 years. The results were comparable to the self-complete version indicating that versions can be used interchangeably.

## 1. Introduction

### Background 

Health-Related Quality of Life (HRQoL) is a multi-faceted and subjective measure of how a person perceives their functional, social, and mental well-being in their environment [1]. HRQoL can be measured using Patient-Reported Outcome Measures (PROMs) by which the descriptive information generated from these PROMs can be used to guide healthcare professionals in tailoring patient interventions [2,3,4] and improving treatment outcomes [3,4]. PROMs can further be used to inform population health, clinical research studies and decision making and health technology assessment [1]. In the past, an emphasis was placed on adult HRQoL, which limited the research available in paediatric populations [5,6,7,8,9]. A contributing factor was the lack of paediatric PROMs, however, with more being developed, a shift to paediatric HRQoL is now being seen [10]. Children who have the cognitive ability to self-report should be encouraged, as far as possible, to do so on valid and reliable PROMs [2].

The EQ-5D-Y-3L is one of approximately 30 generic HRQoL instruments that were developed over the last two decades specifically for the paediatric population [3]. It was adapted from the adult version, the EQ-5D, and measures health across five dimensions namely Mobility (walking about), Looking After Myself (washing and dressing), doing Usual Activities (going to school, hobbies, sports, playing, doing things with family or friends), having Pain or Discomfort and feeling Worried, Sad or Unhappy. The original youth version, EQ-5D-Y-3L, describes health on three levels (no problems, some problems and a lot of problems) which results in 243 (3^5^) health states [4,11]. It further measures general health on a visual analogue scale (VAS) of 0 (worst imaginable health) to 100 (best imaginable health). The first preference-based value sets have been published for Japan and Slovenia [12,13] following the protocol suggested by Romos-Goñi et al. 2020 [14]. The EQ-5D-Y-3L self-complete has been used in studies in South Africa to measure health and changes over time in children from 8 years of age, which is the recommended lower age for self-report [3,4,11,12,13]. 

In South Africa, as of 2016, the Progress in International Reading Literacy Study estimated that 78% of children between the ages of 9 and 10 years had not mastered basic reading by the end of their fourth year of formal schooling compared to a mere 4% internationally [15]. This problem may be more evident in lower socioeconomic settings [16]. It is anticipated that lower literacy levels would directly affect their ability to accurately self-complete any HRQoL instrument despite their age or level of education suggesting otherwise. However, this may not affect their self-report ability if the concepts of health are understood. Furthermore, children with conditions requiring prolonged periods of hospitalization, need for isolation or being too unwell to attend school may be further restricted in their access to schooling and impacted with regards their ability to progress their literacy skills, therefore affecting their reading ability. These children should not be excluded from self-reporting on their health due to difficulties with reading ability. 

Despite the development of approximately 30 generic paediatric PROMs, the modes of administration remain limited to largely self-complete or proxy report [17]. An interviewer-administered version of the EQ-5D-Y-3L has recently been developed by the EuroQoL foundation with a standardised script and instructions for the interviewer. This version has the potential to allow children who struggle to read but are able to cognitively comprehend health-related questions the opportunity to self-report on their health. If this method of administration is successful, it may negate the need for proxy report, which is often the default method of administration despite this mode having been proved to be problematic in some cases with responses often mismatched between children and parents [5,6,7,18]. Studies have found that children and parents prioritise their physical and emotional wellbeing differently and parents are more aware of their child’s physical wellbeing compared to their psychosocial wellbeing, therefore, conflicting information may be reported in these dimensions [19,20]. The aim of this study was thus to determine the performance of the EQ-5D-Y-3L interviewer-administered version compared to the self-complete version in children aged 8–10 years. 

## 2. Materials and Methods

### 2.1. Study Design 

A cross-sectional, descriptive observational, analytical cohort design was conducted in children aged 8–10 years in the Western Cape, South Africa. 

### 2.2. Research Settings

Three research settings, each with children in different health states, but from similar socioeconomic backgrounds (low to middle income), were used in Cape Town, South Africa. Children attending a mainstream school, with generally healthy learners, were used to recruit a general population sample. Children with a functional disability were recruited from three schools for learners with special educational needs. These schools have specialised education services for learners with normal intellect diagnosed with a functional disability (e.g., cerebral palsy, spina bifida or muscle disease). Children with a chronic respiratory illness were recruited at routine outpatient visits at a tertiary paediatric hospital. Children requiring acute medical treatment post fracture or corrective orthopaedic surgery were recruited from the outpatient fracture clinic or the inpatient wards of an acute tertiary paediatric hospital and a paediatric orthopaedic hospital. 

### 2.3. Participants and Sample Size

Participants were included and excluded based on the criteria outlined in Table 1.

The sample size was adequately powered (95%) to detect a difference in correlation and proportion of scores between the three condition groups with a small effect size of 0.4 and a significance of 0.05. 

### 2.4. Instruments

#### 2.4.1. EQ-5D-Y

The official EQ-5D-Y-3L self-complete English version for South Africa was used in this study. The interviewer-administered version for the United Kingdom was tested for equivalence in English for South Africa by the EuroQol group before it was used in this study. The EQ-5D-Y-3L consists of five dimensions namely Mobility (walking about), Looking After Myself (washing and dressing), doing Usual Activities (going to school, hobbies, sports, playing, doing things with family or friends), having Pain or Discomfort and feeling Worried, Sad or Unhappy. Each dimension has three levels of report categorised as level 1 indicating ‘no problems’, level 2 indicating ‘some problems’ or level 3 indicating ‘a lot of problems’ [5]. The EQ-5D-Y-3L includes a VAS which is a vertical, graduated number scale from worst imagined health state (0) to best imagined health state (100) on which the participant rates their overall health status also on the day of testing [6,7]. The EQ-5D-Y-3L has been successfully tested for validity, reliability and responsiveness in South African children aged 8–15 years [11,16,18,21]. As there is no preference-based value set available for South Africa, the recently published value set produced for Slovenia was used [12]. To ensure that the societal preference-based score did not influence performance, comparison was made to the Japanese value set [13]. The Japanese and Slovenian value sets were the only two value sets available at the time of data analysis thus no consideration was given to additional factors such as literacy levels, social structure, etc. 

#### 2.4.2. Faces Pain Scale-Revised (FPS-R) 

The Faces Pain Scale-Revised (FPS-R) is a self-report measure intended to determine the intensity of pain felt by children on the day of testing. It was developed using a series of six facial expressions depicting an increase in pain intensity from left to right. The scoring ranges from 0–10 and increases by increments of 2. It can be used to self-rate pain intensity in children aged four years or older [22]. The FPS-R was used successfully to determine concurrent validity for the dimension of Pain or Discomfort on the EQ-5D-Y-3L in South Africa at baseline [4]. 

#### 2.4.3. Moods and Feelings Questionnaire (MFQ)

The Moods and Feelings Questionnaire (MFQ) consists of 13 questions about the child’s psychological wellbeing in the two weeks before testing. Participants were asked to answer questions on a scale of ‘not true’, ‘sometimes’ or ‘true’. The measure was found valid and reliable in an international study in children from age five years [23].

#### 2.4.4. WeeFIM

The WeeFIM is an observational instrument used to assess functional independence in children [24,25]. Functional performance was measured across three dimensions, namely self-care, mobility and cognition. There is a total of 18 items, each rated on an ordinal scale from 1 to 7. The scale gives scores for sub-scales (mobility, cognition and self-care) or a total score for functional performance; the higher the score, the more independent the child is considered to be in that dimension. The WeeFIM sub-scale of mobility and self-care was previously used to determine concurrent validity in the corresponding dimensions of Mobility and Looking After Myself on the EQ-5D-Y-3L in South Africa [4] and was similarly used in this study.

#### 2.4.5. Preference for the Version

The interviewer captured the child’s preference for the interviewer-administered or self-complete version and the reasoning behind their preference. 

### 2.5. Procedure 

Ethics approval was obtained from the University of Cape Town, Faculty of Health Sciences, Human Research Ethics Committee (HREC 369_2020). The study was carried out following the declaration of Helsinki involving human participants [26] and the recommended COVID precautions and restrictions set out by the local government. 

Information leaflets detailing the study were sent home with eligible learners at the mainstream school and schools for learners with special educational needs. Those parents who were willing, returned signed informed consent and demographic information to school. Children were interviewed in a private room after providing assent. Children attending outpatient clinics were recruited on the day of their routine appointments and those admitted to the inpatient setting were recruited from the ward. All children completed the EQ-5D-Y-3L, FPS-R and MFQ in random order, with the interviewer-administered and self-complete versions separated by the FSP-R, MFQ and the WeeFIM completed by the researcher. 

### 2.6. Data Management and Analysis

The Shapiro-Wilk test was used to test the normality of the data, and as it was not normally distributed, non-parametric tests were used. The level of statistical significance was set at *p* < 0.05. 

#### 2.6.1. General Performance and Feasibility

The EQ-5D-Y-3L responses and descriptive data were summarised in terms of frequency of responses. The feasibility was assessed by comparing the number of missing values for two of the EQ-5D-Y-3L measures. The ceiling effect of the EQ-5D-Y-3L was defined as the proportion of children scoring no problems in a dimension or across all five dimensions (11,111). The floor effect is the proportion of children scoring the most severe problems for a dimension or across all five dimensions (33,333). Differences in reporting were determined via a chi-square statistic (x^2^).

#### 2.6.2. Inconsistent Responses 

Paired dimension responses on the interviewer-administered and self-complete versions were assessed for the respondents who had no missing responses and the proportion of inconsistencies was recorded. 

#### 2.6.3. Known-Group Validity

Known-group validity was tested for the dimensions of the interviewer-administered and self-complete versions for age (continuous variable), sex (male and female) and by health condition (orthopaedic, chronic respiratory illness, functional disability and general population) using Spearman rank-order coefficients (r_s_). It was expected that children with an orthopaedic condition and those with a functional disability would report more problems in the Mobility dimension compared to other groups [7,11,24]. It was also anticipated that children with an orthopaedic condition (being more acutely ill) would report more problems with Usual Activities and Pain or Discomfort [11,25]. Lastly, it was expected that all children with a health condition (orthopaedic, respiratory and functional disability) would report greater feelings of Worried, Sad or Unhappy than children from the general population [11,25]. No difference by age or sex was anticipated. 

The known-group validity across health conditions was assessed for the median utility score and VAS score across the health conditions using the Kruskal Wallis test. It was anticipated that the VAS and utility scores would be higher for those from the general population, functional disability, respiratory condition and orthopaedic condition in that order. 

#### 2.6.4. Concurrent Validity

The Pearson’s correlation of the utility score and VAS score was computed for the EQ-5D-Y-3L self-complete and interviewer-administered versions and compared using the Fisher r-to-z transformation [http://vassarstats.net (accessed on 30 August 2021)]. It was expected that there would be no difference in concurrent validity between the two versions. 

#### 2.6.5. Convergent Validity

Convergent validity between the interviewer-administered and self-complete versions was evaluated using individual dimension response pairs, using Gamma correlations statistics. Utility scores were compared with the Pearson correlation coefficient. Correlation coefficients were interpreted according to Cohen: 0.1–0.29 low association, 0.3–0.49 moderate association and ≥0.5 high association [27]. It was expected that similar dimensions would show similar correlations [11,18]. The convergent validity of the dimension scores of the EQ-5D-Y-3L self-complete and interviewer-administered versions were compared to similar items on the MFQ, FPS-R and WeeFIM using Spearman correlations (r_s_). Correlation coefficients were compared between the versions of the EQ-5D-Y-3L using the Fisher r-to-z transformation [http://vassarstats.net (accessed on 30 August 2021)]. 

#### 2.6.6. Preference between Versions 

Preference between the interviewer-administered and self-complete versions was assessed during cognitive debriefing. It was expected that participants would prefer the interviewer-administered version as the respondent burden was reduced [20].

All data analyses were conducted using SPSS Windows 27.0 (IBM SPSS Inc., Chicago, IL, USA) and Statistica Windows Version 13.0 (TIBCO Software Inc., Palo Alto, CA, USA).

## 3. Results

### 3.1. Recruitment Summary

**Figure 1:**
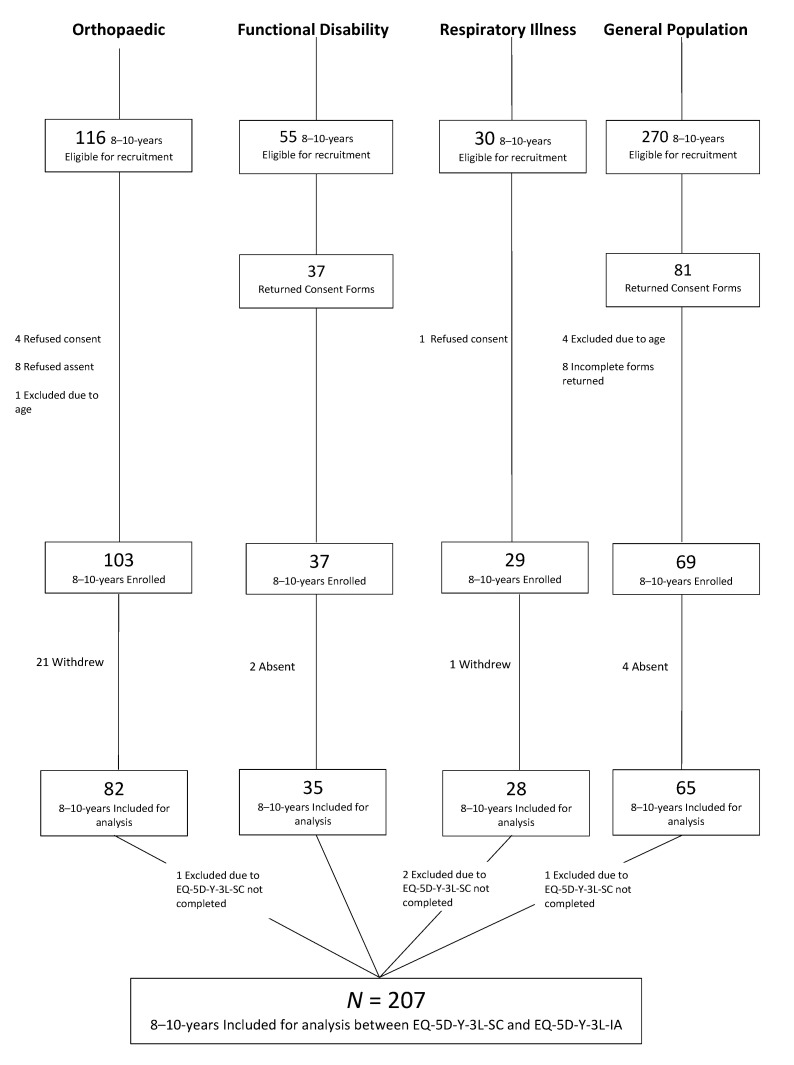
Recruitment of sample.

Figure 1 details the recruitment of children with a total of 207 included for analysis across children known to have an acute orthopaedic condition (*n* = 81), functional disability (*n* = 36), chronic respiratory illness (*n* = 26) or from the school-going general population (*n* = 64). A total of 211 children were recruited, however, only 207 were included in this study as four children did not complete the EQ-5D-Y-3L self-complete. There was a high proportion of non-responders in the 8–10-year-olds (*n* = 260, 55%). Reasons for not responding or refusal of consent/assent was not collected. There was also a high number of children with orthopaedic problems who withdrew (*n* = 21, 20%) during interviews due to personal reasons, transport issues, multiple medical appointments and time constraints. 

### 3.2. Descriptive Statistics of Sample

There was no difference between sex (x^2^ = 0.03, *p* = 0.985) and health condition (x^2^ = 3.61, *p* = 0.729) across 8-, 9- and 10-year-olds (Table 2). In the total group, there were more children with orthopaedic conditions (*n* = 81, 39%) and from the general population (*n* = 64, 31%) than children with functional disabilities (*n* = 36, 13%) and chronic respiratory illnesses (*n* = 26, 13%). The specific conditions included in these disease groups are shown in Table 2. The general population reported relatively few health conditions most frequently including asthma, eczema and allergies (atopy).

### 3.3. General Instrument Performance and Feasibility

Table 3 shows that there were more problems reported across the dimensions of Looking After Myself, Pain or Discomfort and Worried, Sad or Unhappy on the interviewer-administered version compared to self-complete, although these were not significant. The utility score (analysis with the Slovenian utility score is presented and there was no significant difference between results using the Slovenian or Japanese utility scores) and VAS score were similarly higher on the interviewer-administered version, although not significantly so. The ceiling effect was not significantly different between versions either. There were 22 children with missing responses on the self-complete version. The proportion of missing responses across the five dimensions was significantly higher in the 8-year-olds (*n* = 34, 10%) than 9-year-olds (*n* = 14, 4%) or 10-year-olds (*n* = 17, 5%) (x^2^ = 14.23, *p* < 0.001)

Overall, the interviewer-administered version took less time to complete (median = 110 s, IQR = 98, 124 s) compared to the self-complete version (median = 157 s, IQR = 123 s, 209 s). When comparing the time taken across ages, 8-year-olds took the longest to complete both versions but were able to complete the interviewer-administered version quicker than the self-complete version. 

### 3.4. Inconsistency between Interviewer-Administered and Self-Complete Versions

Table 4 shows that the highest report of inconsistent responses between the interviewer-administered and self-complete versions was in the dimension of Pain or Discomfort. The highest inconsistency across dimensions is moving from reporting no problems on the self-complete version and some problems on the interviewer-administered version. An exception to this was the dimension of Worried, Sad or Unhappy and Pain or Discomfort where the highest inconsistency was reporting no problems on the interviewer-administered version but some problems on the self-complete version. 

There were no significant inconsistencies noted by sex (x^2^ = 0.43, *p* = 0.980), age (x^2^ = 2.46, *p* = 0.640) or health condition (x^2^ = 7.8, *p* = 0.801). 

### 3.5. Known-Group Validity

There were no significant differences in rank order correlations of dimension scores for either version (Table 5) by age, sex or health condition. 

**Figure 2:**
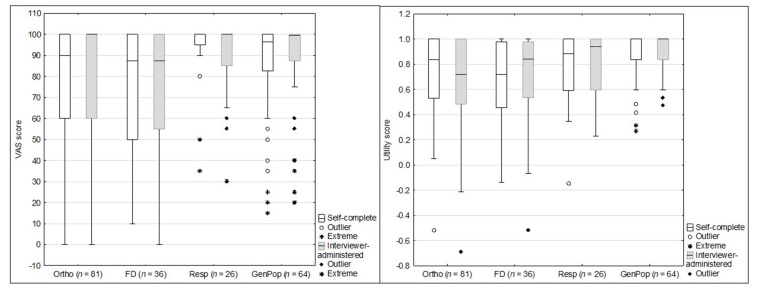
VAS and utility scores for the self-complete and interviewer-administered versions across known health groups. Boxes indicate first (25th) to third (75th) quartiles, the dividing line indicates the median, whiskers indicate the remaining points up to the length of 1.5 times the interquartile range and markers indicate any remaining outliers.

As seen in Figure 2, the utility scores (analysis with the Slovenian utility score is presented and there was no significant difference between results using the Slovenian or Japanese utility scores) were significantly different between groups on the self-complete version (H = 15.84, *p* = 0.001) and interviewer-administered version (H = 26.306, *p* < 0.001). Post-hoc analysis showed that differences on the self-complete version were between those from the general population and children with an acute orthopaedic condition and (H = −3.59, *p* = 0.001) and functional disability (H = −3.135, *p* = 0.002). The interviewer-administered version similarly found differences between the general population and an acute orthopaedic condition (H = 4.939, *p* < 0.001), functional disability (H = −3.252, *p* < 0.001) and additionally those with a chronic respiratory illness (H = −2.124, *p* < 0.001).

The VAS score was significantly different between groups on the self-complete version (H = 15.84, *p* = 0.001) but not the interviewer-administered version (H = 6.59, *p* = 0.086). Post-hoc analysis showed differences on the self-complete version between children with a chronic respiratory illness and functional disability (H = −2.54, *p* = 0.011) and orthopaedic condition (H = 2.626, *p* = 0.009).

### 3.6. Concurrent Validity

**Figure 3:**
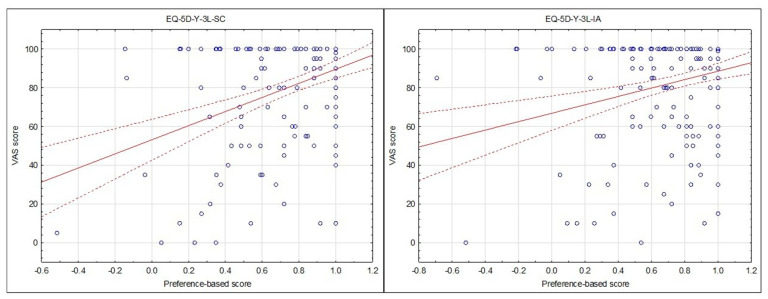
Scatterplot of utility scores versus VAS scores for the EQ-5D-Y-3L Self-Complete (SC) and EQ-5D-Y-3L Interviewer-Administered (IA) versions in children 8–10 years (*n* = 207).

The concurrent validity was assessed via the correlation of the VAS score and utility score (analysis with the Slovenian utility score is presented and there was no significant difference between results using the Slovenian or Japanese utility scores), which was significant and moderate for the self-complete version (r = 0.38, *p* < 0.001) and significant and low for the interviewer-administered version (r = 0.27, *p* < 0.001). There was however no significant difference between the correlations on the interviewer-administered and self-complete versions (z = 1.34, *p* = 0.090) (Figure 3). 

### 3.7. Convergent Validity 

The dimension correlations between the self-complete and interviewer-administered versions were all high and significant. The gamma correlation for the physical dimensions of Mobility, Looking After Myself and Usual Activities showed similar high correlations with Pain or Discomfort and Worried, Sad or Unhappy showing lower correlations when considering all children aged 8–10 years (Table 6). The dimension of Mobility showed a significantly higher correlation than Pain or Discomfort (z = 2.28, *p* = 0.011) and Worried, Sad or Unhappy (z = 1.59, *p* = 0.05). 

The 8-year-olds showed significantly lower correlations than the 10-year-olds in the dimensions of Mobility (z = −2.88, *p* = 0.002), Usual Activities (z = −4.08, *p* < 0.001) and Pain or Discomfort (z = −3.75, *p* < 0.001). The 9-year-olds similarly showed significantly lower correlations than the 10-year-olds for dimensions of Mobility (z = −2.88, *p* = 0.002), Usual Activities (z = −3.17, *p* < 0.001), Pain or Discomfort (z = −2.88, *p* = 0.002) and Worried, Sad or Unhappy (z = −1.97, *p* = 0.020). However, the correlation for Looking After Myself was significantly higher in the 9-year-olds when compared to the 10-year-olds (z = 1.71, *p* = 0.04). 

Both EQ-5D-Y-3L versions showed moderate to high convergent validity with individual items that were hypothesised to show an association and the dimension total scores on the WeeFIM, FPS-R and MFQ (Table 7). The only exception was the dimension of Usual Activities which showed no association with social interaction measured on the WeeFIM but showed low to moderate associations with the physical dimensions of the WeeFIM. There were significantly higher correlations on the WeeFIM and the interviewer-administered versions for items of locomotion, stairs, dressing and the total scores when compared to EQ-5D-Y-3L dimensions of Mobility and Looking After Myself. 

### 3.8. Preference between Versions

There were more 8–10-year-olds who preferred the EQ-5D-Y-3L interviewer-administered version (*n* = 125, 60%) compared to those who preferred the EQ-5D-Y-3L self-complete (*n* = 77, 37%) or had no preference (*n* = 5, 2%) (x^2^ = 21.87, *p* < 0.001). There was no significant difference between preferences for sex (x^2^ = 5.07, *p* = 0.079), age (x^2^ = 5.12, *p* = 0.275) or health conditions (x^2^ = 3.72, *p* = 0.715).

As seen in Table 8 the interviewer-administered version was preferred across all age groups as they reported that they did not yet have the literacy skills for self-completion: “I can’t read yet, I am still learning to read”. This was notably higher in those aged 8–9 years. However, the 10-year-olds did report that they preferred it to the self-complete version as it was easier, quicker, more understandable and factors associated with the interviewer (e.g., from Respondent “you read it nice and slow”), which could all indicate some difficulty with literacy. The general preference included children stating that it was “better” or “nicer”.

The preference for the self-complete version across the age groups was related to independence on completion with children stating, “*I liked to do it on my own*”. General preference for the measure was not specific and included “*I liked it more, it was better*”.

The reason for no preferences included: “both were fine”, “both were easy” or “I liked both”. 

## 4. Discussion

This was the first study to compare the performance of the self-complete and interviewer-administered versions of the EQ-5D-Y-3L. The interviewer-administered version of the EQ-5D-Y-3L proved to be valid by performing as well as the self-complete version in children aged 8–10 years. The feasibility of the instrument is improved with no missing responses on the interviewer-administered version compared to 19% of missing responses on the self-complete version across the five dimensions. Considering the administration in a clinical setting, the interviewer-administered version was however feasible with a relatively low completion time of under three minutes (median = 110 s, IQR = 98 s, 124 s). This is lower than the times reported for self-complete on other generic measures of HRQoL, i.e., Child Health Utility-9D (CHU-9D) (3–5 min), Health Utilities Index (8–10 min), KIDSCREEN (5–20 min) and Paediatric Quality of Life Inventory 4.0 Generic Core Scales (PedsQL) (10–15 min) [27]. The results of the self-complete and interviewer-administered versions are comparable, which would further allow researchers to use the versions inter-changeably in a study and select a version most appropriate to the child’s literacy level and/or medical condition. As the number of missing responses was significantly higher in 8-year-olds, it may be beneficial to have targeted use of interviewer administration in settings where the literacy level may negatively influence self-completion. Based on the reasons for preference of the interviewer-administered version, this sample of children all struggled with literacy skills, and this decreased with the increasing age of the child with 25% of 8-year-olds using literacy skills as their reason followed by 23% in 9-year-olds and 18% in 10-year-olds. The low level of literacy skills may be a problem that is unique to the South African sample recruited, which is reported to have lower literacy levels in this age group compared to international levels [15]. Other reasons for preferring the interviewer-administered version may be associated with acquiescence bias [28], which is mostly associated with interviewer-administered versions rather than self-complete as participants often find it easier to respond with a positive response option, the simplest answer or the first answer [19]. In the context of the EQ-5D-Y-3L, this translates to reporting level one (no problems) therefore presenting with better HRQoL. 

Concurrent validity between the utility and VAS scores were significant for the self-complete and interviewer-administered version (*p* < 0.001) but ranged from low (r = 0.28) to moderate (r = 0.38) in terms of associations. One would expect that the dimensions on the EQ-5D-Y-3L would account for the measure of general health as scored on the VAS and there would be no difference between the self-complete and interviewer-administered descriptive systems. The association between the scores was lower in this study than a previous comparison between the VAS and composite score in children with acute illness (r = −0.786, *p* < 0.001) [20]. Composite scores are a summary of the EQ-5D-Y-3L dimensions using quality-adjusted life year weightings as suggested by Craig et al. (2016) and therefore provide a total score for all five dimensions [29]. It should be noted though that Scott et al. (2017) did not find any association between the composite score and VAS in children with chronic illness or the general population [20]. As this study analysed a heterogenous group of children including those with acute and chronic illness and from the general population it could account for the lower correlation. This could be due to the disability paradox reported in previous studies where children with chronic health conditions, such as cystic fibrosis and functional disabilities, did not necessarily report poorer HRQoL as one would have expected, as children with long-term conditions often find ways to adapt to their environment or the manner in which they complete a task so that it suits their abilities [21,30,31]. Importantly, there was no difference between the utility and VAS scores on the self-complete and interviewer-administered version.

The inconsistency in responses between the interviewer-administered and self-complete versions, although not significant, may be attributed to social desirability bias [32] as face-to-face interviews have been shown to produce more socially desirable responses compared to self-complete versions as participants often feel as though they need to present themselves in the best way when interviewed [33]. Studies comparing these two modes of administration found that self-report instruments were mostly associated with poorer results while face-to-face interviews were associated with more positive results [34]. Similarly, when comparing HRQoL in asthmatic patients, a higher HRQoL was reported on the interviewer-administered version of the instrument [35]. Conversely, no meaningful difference was found between self-report and interviewer-administration when assessing HRQoL in children and adolescents with oral health conditions and adults with acquired immunodeficiency syndrome [36,37]. 

Inconsistencies in this study were most evident with the higher reporting of problems with Worried, Sad or Unhappy and Pain or Discomfort on the self-complete version. This was similarly noted by the lower correlation between Pain or Discomfort and the FPS-R on the self-complete (r_s_ = 0.33) compared to the interviewer-administered version (r_s_ = 0.38) and between Worried, Sad or Unhappy and the MFQ on the self-complete (r_s_ = 0.17–0.33) compared to the interviewer-administered version (r_s_ = 0.16–0.34). Conversely, there was a higher report of problems on the interviewer-administered versions for physical dimensions which may be attributed to observation bias. This may have been further strengthened by the interviewer being a physiotherapist and assessing functional ability on the WeeFIM. A similar observation was seen in a study by Scott et al. (2017) whereby 14% of children reported problems with Mobility which was not observed by the researcher on completion of the WeeFIM. It was found that the report of problems was not only associated with physical impairments but also environmental barriers linked to safety in the areas in which they live [20]. The influence of the interviewer may further be contributing to the significantly higher convergent validity noted with the interviewer-administered dimensions of Mobility and Looking After Myself and the corresponding interviewer-rated WeeFIM items. When looking at the psychosocial dimensions separately, the Worried, Sad or Unhappy dimension on both versions had a moderate association with the MFQ total and showed significant associations with individual items of feeling unhappy, lack of enjoyment during activities of daily living and feeling restless. However, the self-complete version showed slightly stronger associations compared to the interviewer-administered version. This study is the first to use the MFQ as a comparison to the Worried, Sad or Unhappy dimension on either version of the EQ-5D-Y-3L, and therefore, comparisons to other studies were unfortunately not possible. Previous studies have tested convergent validity of the Worried, Sad or Unhappy dimension against psychosocial dimensions on other generic HRQoL instruments such as the KIDSCREEN, PedsQL and CHU-9D and found strong associations between instruments [7,38]. The Pain or Discomfort dimension has previously been compared to the FPS-R, which showed a significant correlation between instruments for acutely ill children only (*p* < 0.001) [20]. Similarly, significant and moderate associations were found in this study between the FPS-R and the interviewer-administered version (r_s_ = 0.33, *p* <0.001) and self-complete version (r_s_ = 0.38, *p* < 0.001) with no significant difference between versions (*p* = 0.281). As a result, this may suggest that the Pain or Discomfort dimension was accurately able to reflect children’s experience of feeling pain and/or discomfort using either version. Assessing psychosocial dimensions remains a challenge due to its subjectivity when compared to physical dimensions such as Mobility, Looking After Myself and Usual Activities, which may be objectively observed [28], therefore, physical dimensions were expected to present with better convergent validity between instruments than psychosocial dimensions. At a dimension level, there was no difference in the ranking by sex, age or health condition, but at a composite level, there were differences in the utility scores between those with and without a health condition on both versions. It is noteworthy that the difference between those with a chronic respiratory illness and the general population was only noted on the interviewer-administered version. This was an expected difference but could not be attributed to any single factor, but it is likely multi-factorial with a difference in reporting of health improved understanding on the interviewer-administered version and/or bias. 

The general population group was from the same geographical catchment area as the tertiary paediatric hospital from where those with a health condition were recruited. The issues found seemed to be reflective of the general population; the results cannot be generalised to the greater Western Cape region as no data on race, home language or socioeconomic status were collected for comparison to the general population of the Western Cape. 

## 5. Conclusions

The EQ-5D-Y-3L interviewer-administered version is valid and reliable for use in children aged 8–10 years. The results were comparable to the self-complete version indicating that versions can be used interchangeably based on the ability of the child instead of defaulting to proxy report if the child is unable to self-complete the PROM. 

The feasibility of the interviewer-administered version is supported by the lack of missing responses; therefore, the burden of interview administration, with regards to increased time and resources [36,39], may be outweighed by the benefit of reduction in missing responses. 

Further studies are recommended to assess whether social desirability bias significantly impacts the reporting of Worried, Sad or Unhappy and Pain or Discomfort in children with conditions that are hypothesised to impact these dimensions, i.e., children experiencing anxiety and/or depression and children with acute pain. 

## Figures and Tables

**Table 1:** Inclusion and exclusion criteria.

Inclusion Criteria	Exclusion Criteria
Children aged 8–10 years.	Children who were medically diagnosed as unable to hear with assistive technology as the primary outcome measure relied on interviewer administration.
Children fluent in English—determined if it was their self-classified home language or the language of instruction at school.	Children attending schools for leaners with special educational needs, with moderate to severe intellectual disability, diagnosed by a psychologist and typically educated in a unit class, as their level of understanding of the questions asked could have been limited. Teachers and school psychologists at the these schools identified children with moderate to severe intellectual disabilities.
Children with multi-morbidities—allocation to a known group was done according to the condition that they were seeking care for on the day of recruitment and any additional health conditions were noted.	Children who required admission to the intensive care or high care unit, with continuous monitoring, were considered critically ill and were excluded to prevent additional associated emotional stress that may occur if they were to participate.
Children attending schools who provided both consent and assent.	

**Table 2:** Descriptive statistics of participants aged 8–10 years.

	Age (Years)							
	8 Years		9 Years		10 Years		Total	
	*n*	%	*n*	%	*n*	%	*n*	%
Sex	(*n* = 65)		(*n* = 70)		(*n* = 72)		(*n* = 207)	
Female	30	46%	32	46%	34	47%	96	46%
Male	35	54%	38	54%	38	53%	111	54%
Orthopaedic	(*n* = 29)		(*n* = 25)		(*n* = 27)		(*n* = 81)	
Upper Limb Fracture	13	45%	9	36%	9	33%	31	38%
Lower Limb Fracture	6	21%	10	40%	6	22%	22	27%
Surgical correction of acquired or congenital orthopaedic condition ^#^	5	17%	4	16%	10	37%	19	23%
Other *	5	17%	2	8%	2	7%	9	11%
Functional Disability	(*n* = 11)		(*n* = 12)		(*n* = 13)		(*n* = 36)	
Developmental Co-ordination Disorder ꭞ	6	55%	8	67%	7	54%	21	58%
Cerebral Palsy	1	9%	2	17%	3	23%	6	17%
Spina Bifida	2	18%	1	8%	2	15%	5	14%
Developmental Delay	1	9%	1	8%	1	8%	3	8%
Traumatic Brain Injury	1	9%	0	0%	0	0%	1	3%
Respiratory	(*n* = 7)		(*n* = 7)		(*n* = 12)		(*n* = 26)	
Atopy	3	43%	2	29%	7	58%	12	46%
Cystic Fibrosis	2	29%	2	29%	1	8%	5	19%
Bronchiectasis	0	0%	0	0%	2	17%	2	8%
Other ^¥^	2	29%	3	43%	2	17%	7	27%
General Population	(*n* = 18)		(*n* = 26)		(*n* = 20)		(*n* = 64)	
None	16	89%	21	81%	16	80%	53	83%
Atopy	1	6%	5	19%	2	10%	8	13%
Other ^§^	1	6%	0	0%	2	10%	3	5%

^#^ Includes Blount’s disease, osteogenesis imperfecta, developmental dysplasia of the hip, leg, length discrepancy and spinal deformity; * includes osteitis, septic arthritis and traumatic amputation; ꭞ includes learning disability and Human Immunodeficiency Virus; ^¥^ includes damage to the lungs post-acute viral infection, congenital abnormalities of the respiratory system and idiopathic pulmonary haemorrhage; ^§^ includes osteogenesis imperfecta and a congenital cardiac defect.

**Table 3:** Comparison of the self-complete and interviewer-administered dimension responses.

		Self-Complete		Interviewer Administered			
		(*n* = 207)		(*n* = 207)			
		*n*	%	*n*	%	X^2^	*p*-Value
Mobility	No	151	73%	146	71%	3.11	0.211
	Some	30	14%	43	21%		
	A lot	12	6%	18	9%		
	Missing	14	7%	0	0%		
Looking After Myself	No	143	69%	150	72%	0.98	0.613
	Some	37	18%	47	23%		
	A lot	12	6%	10	5%		
	Missing	15	7%	0	0%		
Usual Activities	No	145	70%	141	68%	3.79	0.150
	Some	29	14%	47	23%		
	A lot	18	9%	19	9%		
	Missing	15	7%	0	0%		
Pain or Discomfort	No	122	59%	133	64%	0.81	0.667
	Some	56	27%	60	29%		
	A lot	18	9%	14	7%		
	Missing	11	5%	0	0%		
Worried, Sad or Unhappy	No	135	65%	147	71%	0.40	0.819
	Some	52	25%	49	24%		
	A lot	10	5%	11	5%		
	Missing	10	5%	0	0%		
11111		80	39%	62	30%	3.1	0.078
Utility score *	Median (IQR)	0.883 (0.608,1.00)		0.870 (0.614,1.00)		z = 1.262	0.207
VAS *	Median (IQR)	95 (68,100)		100 (70,100)		z = 0.496	0.62

* Difference in continuous variables were calculated with Wilcox sign test.

**Table 4:** Inconsistent responses across dimensions on the self-complete and interviewer-administered dimension versions.

	Interviewer-Administered							
Self-Complete	No		Some		A Lot		Inconsistent Responses	
Mobility	*n*	%	*n*	%	*n*	%	*n*	%
No	122	66%	15	8%	7	4%	41	22%
Some	8	4%	17	9%	3	2%		
A lot	4	2%	4	2%	4	2%		
Looking After Myself	No		Some		A lot			
No	119	65%	16	9%	3	2%	42	23%
Some	13	7%	20	11%	2	1%		
A lot	4	2%	4	2%	3	2%		
Usual Activities	No		Some		A lot			
No	113	61%	18	10%	7	4%	45	24%
Some	7	4%	18	10%	3	2%		
A lot	5	3%	5	3%	8	4%		
Pain or Discomfort	No		Some		A lot			
No	96	52%	20	11%	3	2%	57	31%
Some	15	8%	29	16%	6	3%		
A lot	9	5%	4	2%	2	1%		
Worried, Sad or Unhappy	No		Some		A lot			
No	110	60%	15	8%	4	2%	48	26%
Some	21	11%	24	13%	3	2%		
A lot	3	2%	2	1%	2	1%		

*n* = 184, shaded cells indicate consistent responses.

**Table 5:** Spearman’s rank correlation of self-complete and interviewer-administered scores across health groups, age and sex.

	Age * (Years)		Sex		Health Condition ^#^	
	Self Complete	Interviewer Administered	Self Complete	Interviewer Administered	Self Complete	Interviewer Administered
Mobility	0.03	−0.01	−0.01	0.09	0.04	0.00
Looking After Myself	−0.02	−0.04	0.07	0.06	0.02	−0.09
Usual Activities	−0.07	0.01	−0.07	0.10	0.01	0.03
Pain or Discomfort	−0.08	0.05	0.04	0.13	0.04	−0.10
Worried, Sad or Unhappy	0.02	−0.02	−0.04	0.05	0.13	0.00

* Age was computed as a continuous variable. ^#^ Health condition was compared by those with an orthopaedic condition, functional disability, chronic respiratory illness and the general population.

**Table 6:** Gamma correlations of self-complete and interviewer-administered dimension responses.

Self-Complete	Interviewer-Administered				
	Mobility	Looking after Myself	Usual Activities	Pain or Discomfort	Worried, Sad or Unhappy
Mobility	0.74 *	0.18	0.52 *	0.55 *	0.19
Looking After Myself	0.58 *	0.76 *	0.59 *	0.20	0.32 *
Usual Activities	0.51 *	0.46 *	0.75 *	0.31 *	0.21 *
Pain or Discomfort	0.44 *	0.41 *	0.43 *	0.62 *	0.47 *
Worried, Sad or Unhappy	0.44 *	0.39 *	0.28 *	0.52 *	0.66 *

*n* = 207; * *p* < 0.05.

**Table 7:** Convergent validity of the self-complete and interviewer-administered version and corresponding items in the WeeFIM, Faces Scale-Revised and Moods and Feelings Questionnaire.

	EQ-5D-Y-3L			
	SC	IA	SC vs. IA	
			z-Score	*p*-Value
WeeFIM Mobility	Mobility			
Locomotion (walk/wheelchair for ≥45 m OR crawl ≥15 m)	−0.31 **	−0.45 **	1.66	0.049
Stairs climbing (ascend and descend 12–14 stairs)	−0.23 **	−0.47 **	2.79	0.003
Motor Total	−0.23 **	−0.40 **	1.91	0.028
WeeFIM Self-Care	Looking After Myself			
Grooming	−0.25 **	−0.39 **	1.58	0.057
Bathing (washing body excluding back)	−0.45 **	−0.68 **	3.48	0
Dressing Upper Body	−0.38 **	−0.59 **	2.69	0.004
Dressing Lower Body	−0.43 **	−0.62 **	2.68	0.004
Self-Care Total	−0.44 **	−0.66 **	3.24	0.001
WeeFIM Mobility	Usual Activities			
Mobility Total	−0.29 **	−0.48 **	2.27	0.012
Motor Total ^§^	−0.23 **	−0.40 **	1.91	0.028
WeeFIM Cognition				
Social Interaction (interaction with other children)	0.04	0.05	−0.1	0.4602
	Pain or Discomfort			
Faces Pain Scale-Revised	0.33 **	0.38 **	−0.58	0.281
Moods and Feelings Questionnaire	Worried, Sad or Unhappy			
Unhappy	0.26 **	0.18 **	−0.58	0.281
Enjoyment	0.21 **	0.19 **	0.21	0.417
Restless	0.21 **	0.19 **	0.21	0.417
No good	0.21 **	0.16 *	0.52	0.302
Crying	0.17 *	0.22 **	−0.53	0.298
Total	0.33 **	0.34 **	−0.11	0.456

N = 207, SC = self-complete, IA = interviewer-administered. ^§^ Motor Total = Mobility total + Self-care total. * Spearman’s correlation *p* < 0.05, significant z scores are bolded. ** Spearman’s correlation *p* < 0.001. A higher Moods and Feelings score, Faces Pain Scale-Revised score and EQ-5D-Y-3L score all indicate greater problems. A higher WeeFIM score indicates greater independence.

**Table 8:** Reason for preference between the self-complete and interviewer-administered versions.

	8-Years		9-Years		10-Years		Total	
	(*n* = 65)		(*n* = 70)		(*n* = 72)		(*n* = 207)	
Reason for interviewer-administered preference	*n*	%	*n*	%	*n*	%	*n*	%
Associated with literacy skills	16	25%	16	23%	13	18%	45	22%
Easier	5	8%	6	9%	4	6%	15	7%
Quicker	2	3%	0	0%	0	0%	2	1%
More understandable	4	6%	8	11%	7	10%	19	9%
Enjoyed the conversation	0	0%	1	1%	1	1%	2	1%
Listening/answering preferred over reading	4	6%	2	3%	6	8%	12	6%
Associated with interviewer	3	5%	3	4%	8	11%	14	7%
General preference	3	5%	4	6%	3	4%	10	5%
Other ^#^	1	2%	4	6%	1	1%	6	3%
Reason for self-complete preference								
Associated with independence	20	31%	16	23%	19	26%	55	27%
General preference	4	6%	3	4%	1	1%	8	4%
More understandable	1	2%	2	3%	1	1%	4	2%
Easier	1	2%	1	1%	1	1%	3	1%
No talking required	0	0%	1	1%	1	1%	2	1%
More time to think about answers	0	0%	0	0%	2	3%	2	1%
Other *	0	0%	2	3%	1	1%	3	1%
No preference	0	0%	1	1%	4	6%	5	2%

^#^ Included the ability to complete the interviewer-administered version, provide the correct answers and feeling nervous to complete the interviewer-administered version and the lack of enjoyment associated with reading. * Included the preference of reading over listening, the self-complete version was quicker and the instructions were clear.

## Data Availability

The data presented in this study are available on request from the corresponding author. The data are not publicly available as per the ethical permission obtained so as to ensure anonymity and confidentiality of participants and their information.
